# Effects of Functional Electrical Stimulation Combined With Usual Physiotherapy on Hand Pain and Function in Stroke: Protocol for a Double‐Blind Randomized Controlled Trial

**DOI:** 10.1002/hsr2.73276

**Published:** 2026-09-23

**Authors:** Ehsanur Rahman, Saiba Muhammad Sabrin, Md. Feroz Kabir, Md. Obaidul Haque, Md. Waliul Islam, Md. Zahid Hossain, Md. Tofajjal Hossain, Kazi Md Azman Hossain, Hassan M. Al‐ Emran

**Affiliations:** ^1^ Department of Physiotherapy and Rehabilitation Jashore University of Science and Technology Jashore Bangladesh; ^2^ Department of Biomedical Engineering Jashore University of Science and Technology Jashore Bangladesh; ^3^ Department of Physiotherapy Centre for the Rehabilitation of the Paralysed (CRP) Dhaka Bangladesh

**Keywords:** functional electrical stimulation, hand function, hand pain, physiotherapy, stroke

## Abstract

**Background and Aims:**

Stroke is a leading cause of long‐term disability, often causing upper extremity paralysis that limits daily activities. Functional electrical stimulation (FES) has been shown to promote motor recovery by activating affected muscles, but its effectiveness when combined rigorously with usual physiotherapy treatment (UPT) remains underexplored. This trial aims to assess whether FES combined with UPT improves hand pain and function in stroke patients more than UPT with placebo FES.

**Methods:**

This prospective, randomized controlled trial will recruit 140 stroke patients, who will be randomly assigned to either the intervention group (*n* = 70) or the control group (*n* = 70). The intervention group will receive 30 min of FES applied to the wrist extensors combined with 30 min of UPT, while the control group will receive placebo FES along with the same UPT protocol, 3 days a week for 8 weeks. Outcomes will be measured at baseline, mid‐intervention (week 4), post‐intervention (week‐8), and after 12‐week follow‐up (week‐20). Primary outcomes will be hand pain, assessed with the Numerical Pain Rating Scale (NPRS), and upper extremity function, assessed with the Fugl‐Meyer Assessment–Upper Extremity (FMA‐UE). Secondary outcomes will include muscle tone, measured by the Modified Ashworth Scale (MAS), and manual dexterity, measured by the Box and Block Test (BBT).

**Results:**

All outcomes will be assessed at baseline, mid‐intervention, post‐intervention, and follow‐up to evaluate both short‐term and sustained effects. Comparative analyses will be conducted to determine the effectiveness of FES combined with UPT versus placebo FES with UPT in improving pain and functional outcomes in stroke.

**Conclusions:**

This trial will provide high‐quality evidence regarding the adjunctive role of FES in stroke rehabilitation. The findings are expected to support evidence‐based physiotherapy practice, enhance clinical decision‐making, and improve functional recovery and quality of life for individuals with stroke‐related upper extremity impairments.

**Trial Registration:** CTRI/2025/07/091676.

## 1 Introduction

Globally, stroke is one of the most frequent causes of death and a primary cause of acquired adult disability [1]. Nearly 12 million new strokes occur annually worldwide. One out of every four people who are above 25 years old will have a stroke in their lives [2]. In Bangladesh, the prevalence of stroke is 1.04 per 1000 people, with males having a significantly higher rate, 1.58 times greater than females [3]. The functional and disability status of individuals post‐stroke varies according to their age, gender, and type, phases, and chronicity of stroke [4]. The upper extremity is one of the most commonly affected stroke areas, with impairments specifically predominant in the hand [5]. Upper extremity disability commonly occurs in about 50% of stroke cases [6, 7, 8]. Patients with stroke tend to experience poor hand performance because of the severity of motor loss, sensory loss, and lack of coordination. Patients with a Fugl‐Meyer upper‐extremity test score below 11 points 2 weeks after stroke have minimal chances of regaining any measurable hand dexterity, as well as a low probability of regaining upper‐extremity functional capacity [9, 10]. Immediate rehabilitation is strongly advised to support individuals who have experienced a stroke in diminishing residual functional disabilities and complications in post‐stroke. Rehabilitation helps to augment the quality of life by reducing functional disability and stroke complications that may mitigate the need and expense of long‐term care [11].

Pain after stroke can impair psychological well‐being, daily function, working abilities, and a person's quality of life [12, 13, 14]. The prevalence of post‐stroke pain ranges from 19% to 74%, influenced by pain classification systems, follow‐up duration, and assessment methods. Central pain can result from thalamic lesions, while peripheral pain can result from secondary deficits and abnormal muscle tone [15]. After a stroke, the upper extremities are the most commonly affected site of peripheral pain. However, hand pain is reported in 4.6% of the elderly stroke population [16, 17, 18]. Functional status of the upper extremity is a significant focus during rehabilitation for people with a stroke [19]. Over the last 10 years, the number of studies focusing on stroke rehabilitation of the upper extremity has increased rapidly [18]. Numerous studies have evaluated the effectiveness of physiotherapy interventions for stroke by comparing experimental treatments with standard or conventional therapy [18, 19, 20, 21]. A variety of therapeutic approaches have been used to improve upper‐extremity motor function following a stroke, including Functional Electrical Stimulation (FES) [9, 10, 18, 19, 20]. FES involves the use of electrical impulses that stimulate the nerves attached to paralysed muscles. It can be employed as an intervention during upper extremity rehabilitation because it stimulates the contraction of paralysed and weak muscles, enabling a person to perform movements and actions such as gripping a key, holding a toothbrush, and using utensils [20]. FES can be used to create movements or functions that resemble typical voluntary movements for the purposes for which those movements are intended [21, 22].

Despite the widespread use of FES in neurorehabilitation, current evidence regarding its effectiveness in reducing hand pain and improving function in people with stroke remains inconclusive. Several studies have reported improvements in motor performance following FES application; however, considerable variability in intervention protocols, outcome measures, and study populations limits the generalizability of the findings. Moreover, pain is a significant yet often underreported barrier to functional recovery and has not been adequately addressed in most of the FES‐focused trials. Notably, few randomized controlled trials (RCTs) have concurrently examined both pain and hand‐specific functional outcomes following FES in individuals with stroke [21]. Although recent literature encourages the use of FES in upper extremity stroke rehabilitation, its application, especially when combined with well‐structured standard care for post‐stroke hand impairments, remains limited and still unfamiliar in clinical settings. Previous studies have shown mixed results, often hindered by poor methodology, limited outcome measures, and a lack of long‐term follow‐up, leaving their optimal effectiveness uncertain when assessed with robust methodological standards [13, 18].

Therefore, this prospective, randomized controlled trial (RCT) is relevant and aims to complement and contribute to the existing scientific effort by maintaining a strong methodological rigor. The primary objective of this trial is to evaluate the effectiveness of FES‐integrated UPT in reducing hand pain and improving hand function in stroke patients. The secondary objectives are: (1) to evaluate sociodemographic factors such as age, gender, stroke type, and time since stroke that may affect post‐stroke pain and functional recovery; (2) to establish baseline compatibility between the experimental and control groups; (3) to assess changes and compare group differences across all outcome measures at mid and post‐intervention periods; and (4) to explore the long‐term benefits of FES‐integrated UPT in maintaining improvements during the follow‐up period. It is hypothesized that FES‐integrated UPT will result in significantly greater improvements in hand pain, function, muscle tone, and manual dexterity compared to placebo FES with UPT, with these improvements being more long‐lasting.

## 2 Methods

### 2.1 Study Design

**Figure 1:**
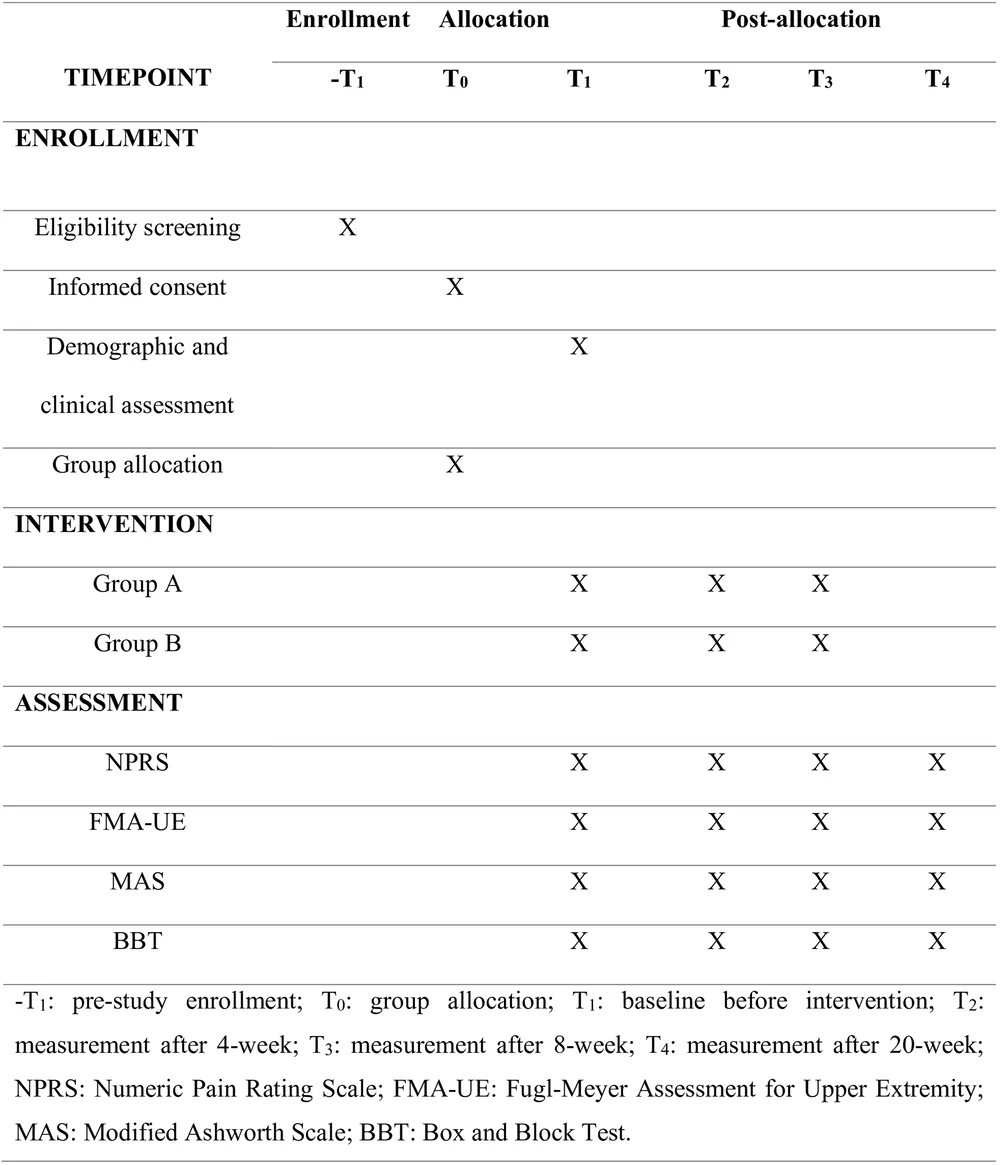
Timeline of enrollment, assessment, and intervention.

**Figure 2:**
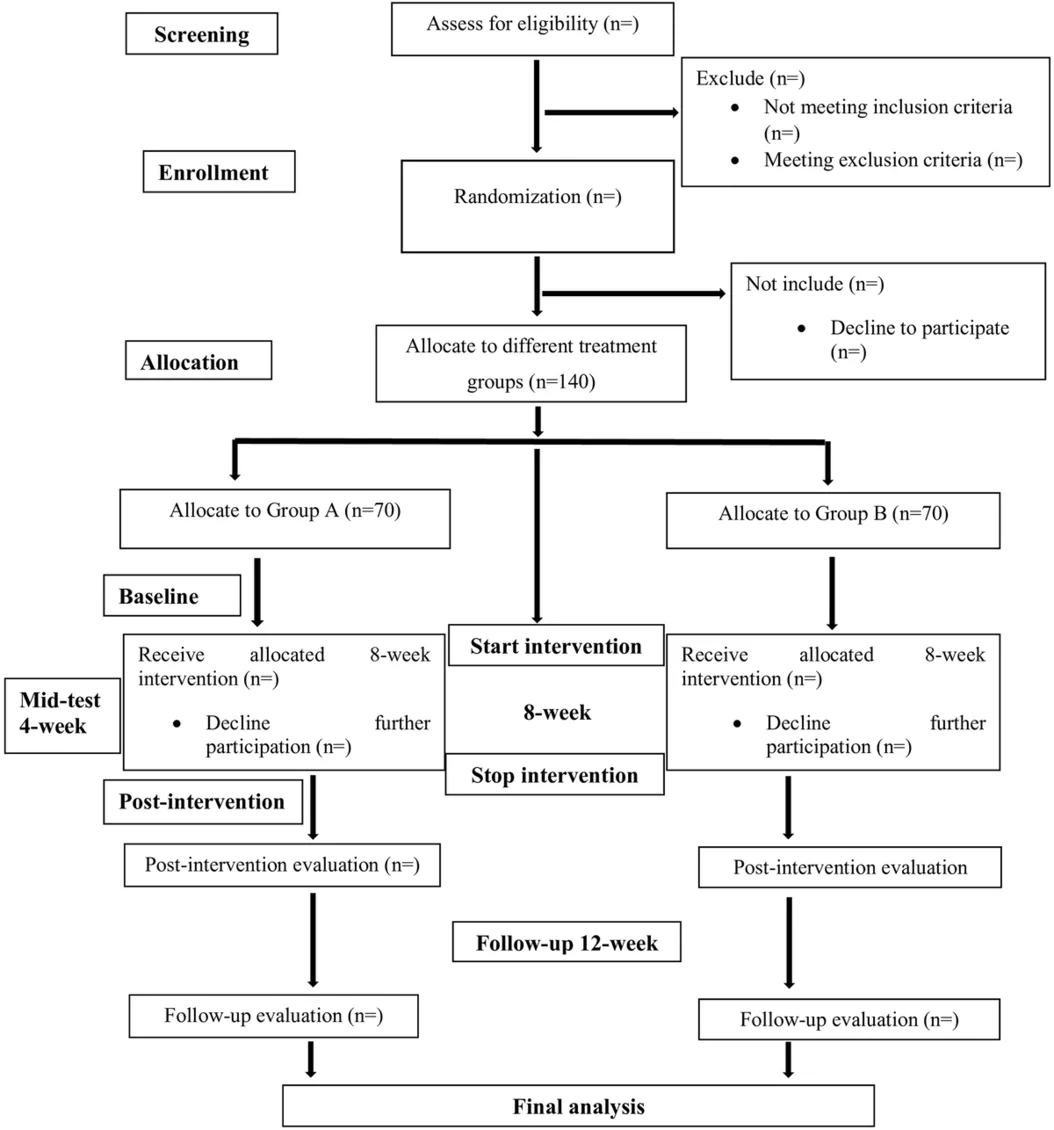
CONSORT flow diagram.

A two‐arm, parallel‐group, randomized, participant‐ and assessor‐blinded controlled trial will be conducted in Bangladesh from November 2025 to April 2026, following the recommendations of the Standard Protocol Items: Recommendations for Interventional Trials (SPIRIT; Figure 1) [22]. Following consent, Randomization will be done among the participants in a ratio of 1:1 by an Excel randomizing procedure to receive either FES with UPT (Experimental group or Group A) or placebo FES with UPT (Control group or Group B) for an 8‐week intervention period. The results will be reported in accordance with the CONSORT (Consolidated Standards of Reporting Trials) guidelines (Figure 2).

### 2.2 Study Settings

This study will involve patients from the Neurology unit of the Physiotherapy and Rehabilitation department of Jashore University of Science and Technology in Bangladesh. This study setting is well‐recognized in Bangladesh for its physiotherapy and rehabilitation services.

### 2.3 Sample Size Calculation

Sample size calculation is performed using G*Power software version 3.1.9.7 for a two‐tailed independent‐groups test comparing two group means. The effect size (Cohen's *d*) is set to 0.5 (medium effect) based on prior research in post‐stroke rehabilitation [23]. The α value is predetermined as 0.05, and the power (1–β) is 0.80 to limit the risk of Type II error. Based on these parameters, we will need 64 individuals per group, for a total of 128 participants. Finally, we plan to recruit 140 participants (70 per group), allowing for an anticipated dropout rate of approximately 10%.

### 2.4 Participants' Recruitment Process

Participant recruitment will begin in November 2025, targeting individuals undergoing post‐stroke rehabilitation who meet predefined inclusion and exclusion criteria. Eligibility assessments will be conducted by trained research assistants who are independent of the rehabilitation process to reduce bias. Each participant will be assigned a unique identifier and coded in an Excel database. Eligible participants will be comprehensively informed of the study's objectives, procedures, risks, and benefits and will provide written informed consent using an institutional ethical review board‐approved form. Then, they will be assigned to a group to receive intervention as per protocol. All study documentation will be securely stored to protect data integrity.

### 2.5 Randomization and Blinding Procedures

Following consent, participants will be randomly assigned in a 1:1 ratio to either Group A or Group B using simple randomization performed through Excel's = RAND() function by an independent research assistant not involved in recruitment, treatment, or assessment. Allocation concealment will be ensured using sequentially numbered, opaque, sealed envelopes prepared by an independent assistant. This study will employ participants, and outcome assessors will be blinded to group allocation throughout the study period. Due to the nature of the intervention, treating physiotherapists administering FES cannot be blinded; however, they will not participate in outcome assessment or data analysis. Participants in both groups will receive identical treatment durations, therapist interactions, electrode placement procedures, and device preparation to maintain the credibility of blinding. Outcome assessments and statistical analyses will be conducted by independent personnel blinded to treatment allocation. Participants will also be instructed not to discuss their intervention experiences with assessors to minimize detection bias.

### 2.6 Eligibiity Critetia

#### 2.6.1 Inclusion Criteria

Participants must meet all of the following to be eligible for the study: (1) Confirm diagnosis of unilateral ischemic stroke verified by CT or magnetic resonance imaging (MRI) by a neurologist [24]; (2) Age between 40 and 70 years; (3) Stable vital signs with normal levels of consciousness; (4) Brunnstrom recovery stage between 3 or less for the affected upper extremity function [25, 26]; (5) Presence of mild‐to‐moderate spasticity in the affected upper extremity, based on Modified Ashworth Scale (MAS) score of 1 to 2 in the wrist flexor muscles; (6) Stroke onset between 1 and 3 months before enrollment [27]; (7) Willingness and ability to provide informed consent to participate in the study.

#### 2.6.2 Exclusion Criteria

Participants will be excluded if they: (1) Have a previous history of stroke, including reversible ischemic or hemorrhagic stroke; (2) Exhibit severe cognitive impairments or significant speech and communication difficulties; (3) Have a diagnosis of any mental health disorders (e.g., schizophrenia); (4) Have an implanted cardiac pacemaker [28].

### 2.7 Interventions

#### 2.7.1 Group A (FES‐Integrated UPT)

This group will undergo treatment consisting of 30 min of FES and 30 min of UPT for 8 weeks, three times weekly. The intervention frequency of three sessions per week was selected based on previous upper‐extremity FES rehabilitation studies demonstrating functional improvements with similar training schedules [18]. Training will be provided to the physiotherapists on the FES application before the intervention begins. The XFT‐2003EA H_2_ Hand Rehab System device will be used to deliver electrical stimulations, which can be driven up to 60 mA amplitude, pulse width 250 µs, pulse frequency 40 Hz [29], to the target wrist extensor muscles according to the individual's tolerance. Participants and caregivers will be given a standard warning about the use of electrical stimulation, including watching for signs of skin burns. The electrodes of FES will be placed on the extensor carpi radialis longus and brevis, and the extensor carpi ulnaris [30]. FES will involve performing wrist extension–facilitated functional activities such as Cylindrical, Spherical, Hook, Pinch, and Thumb Adduction, focusing on power and precision. These tasks were selected because effective wrist extension is biomechanically essential for optimizing finger flexor length‐tension relationships, improving grasp stability, and facilitating functional hand use during upper‐extremity activities.

The UPT will be divided into two equal parts: (1) Spasticity management (15 min), involving passive range‐of‐motion (PROM) exercises to replicate joint movements and elongate muscles and soft tissues in segments without active movement, along with sensory stimulation techniques such as brushing and icing to modulate spasticity of the affected wrist joint during wrist flexion, extension, and rotation. (2) Active/functional upper extremity training (15 min), including hand opening/closing, gripping practice, and wrist extension/flexion tasks, with therapist‐assisted facilitation when residual movement is present, followed by relaxation techniques. All exercises will be performed at a moderate intensity (targeting 10–15 repetitions per task, adjusted to patient tolerance) to promote neuromuscular engagement and prevent fatigue [8, 9, 27]. Each session will combine FES and UPT, and each participant will receive a total of 24 treatment sessions.

#### 2.7.2 Group B (Placebo FES‐Integrated UPT)

This group will receive 30 min of placebo FES combined with 30 min of UPT, three times weekly for 8 weeks, identical to Group A in treatment duration and therapist interaction. For placebo FES, electrodes will be positioned identically to those in the active FES group over the wrist extensor muscles; however, the stimulation intensity will be set below the motor threshold and gradually reduced after a brief initial sensory stimulation period, thereby preventing visible muscle contraction. This approach is intended to maintain participant blinding and enhance placebo credibility while avoiding therapeutic neuromuscular effects. Each participant will receive a total of 24 treatment sessions [18].

To ensure intervention fidelity and minimize performance bias, all physiotherapists involved in treatment delivery will complete training sessions before participant enrollment. A detailed intervention with describing electrode placement, stimulation parameters, task progression, and UPT procedures will be used throughout the study. Treatment adherence will be monitored using structured session checklists completed after each session to ensure protocol compliance and consistency across therapists and participants.

### 2.8 Outcome Measurements

The primary outcome measures will focus on hand pain and function, with hand pain measured using the Numeric Pain Rating Scale (NPRS) and hand function using the Fugl‐Meyer Assessment for Upper Extremity (FMA‐UE). Secondary outcomes will include muscle tone and manual dexterity, with muscle tone measured using the MAS and manual dexterity using the Box and Block Test (BBT). Spasticity will specifically be evaluated in the wrist flexors, finger flexors, and elbow flexors of the affected limb, as these muscle groups are commonly associated with post‐stroke upper extremity spasticity patterns. Individual participant sociodemographic information related to stroke will also be collected at baseline assessment. The primary and secondary outcomes will be measured at four time points: baseline, mid‐intervention (week 4), post‐intervention (week 8), and 12‐week follow‐up (week 20).

### 2.9 Primary Outcomes

#### 2.9.1 Numeric Pain Rating Scale (NPRS)

The NPRS will be used to measure hand pain. It is a reliable and valid 11‐point scale (0–10) measuring pain intensity, where 0 indicates no pain and 10 represents the worst imaginable pain. It is commonly used in healthy adults and patients with various pain conditions, including stroke. The NPRS demonstrates excellent reliability for stroke pain assessment (Cronbach's alpha ≥ 0.95; reliability > 0.95). It is quick to administer (approximately 1 min) and can be completed verbally or graphically [31].

#### 2.9.2 Fugl‐Meyer Assessment for Upper Extremity (FMA‐UE)

The FMA‐UE will be used to measure hand function. It is a widely used, reliable procedure for evaluating motor function in post‐stroke hemiplegia, with reliability and internal consistency values ≥ 0.95 [32]. For the upper extremity, the total value is 66. It uses a 3‐point scale (0–2) to assess motor function, with scores interpreted as follows: < 33: Severe, 33–55: Evident, 56–62: Moderate, 63‐65: Mild motor dysfunction [33].

### 2.10 Secondary Outcomes

#### 2.10.1 Modified Ashworth Scale (MAS)

The MAS will be used to measure muscle tone in the affected upper extremity. It is a valid method for measuring muscle tone, where passive joint movement is scored from 0 (no increase in tone) to 4 (rigidity). The MAS shows good inter‐ and intra‐rater reliability (ICC = 0.61–0.91), though the 1+ grade may present some classification challenges [34].

#### 2.10.2 Box and Block Test (BBT)

The BBT will be used to assess manual dexterity and provide a clearer understanding of hand function. It is a valid test for measuring manual dexterity and hand‐eye coordination. Participants will transfer wooden blocks between compartments within 1 min, with the total number of blocks moved constituting the score. The BBT has excellent validity, reliability (ICC = 0.93–0.98), and sensitivity to changes in upper extremity function post‐stroke [35, 36].

### 2.11 Study Procedure

After recruitment, participants will receive a participant information sheet, and written informed consent will be obtained. Medical screening and physical assessment will then be conducted by trained assessors under the supervision of a physiotherapist. Baseline socio‐demographic and clinical data will be collected by a blinded assessor. Allocation to intervention groups will be concealed using sealed envelopes. Interventions will be provided free of charge, with participants responsible only for transportation costs. After 4 weeks of intervention, participants will be assessed for mid‐intervention measurements. Post‐intervention, participants will return to the assessor for outcome evaluation and receive follow‐up instructions. Follow‐up assessments will be scheduled by phone for the final in‐person evaluation. All study procedures will strictly adhere to the protocol approved by the Ethics Committee. Any deviations required due to emergency situations or unforeseen circumstances will be undertaken only after obtaining prior approval from the Ethics Committee.

### 2.12 Data Management and Monitoring

All study data will be managed in accordance with a standardized Manual of Operations and Case Report Forms (CRFs) to ensure uniformity in recruitment, assessment, data entry, and confidentiality. Staff will be trained in Good Clinical Practice, with hard copy data stored securely in locked cabinets and identifiers kept separately, while electronic data will be maintained in a secure database at the Biomedical Engineering Department, Jashore University of Science and Technology, accessible only to the principal investigator and authorized research staff. Any use of secondary data will require approval from the Data Management Committee. Monitoring will be conducted by two independent personnel not involved in the intervention, who will oversee participant enrollment, protocol adherence, adverse events, and interim analyses. Any necessary modifications to the study design or intervention will be promptly reported to the Ethical Review Board by the principal investigator.

### 2.13 Data Analysis

All analyses will be conducted by an independent statistician using SPSS version 22 with anonymized, encrypted data. Data will be screened for completeness, missing values, and outliers prior to analysis. Normality will be assessed using the Kolmogorov–Smirnov and Shapiro–Wilk tests, along with measures of skewness and kurtosis, and visual inspection (histograms and *Q*–*Q* plots). Descriptive statistics will be presented as mean ± standard deviation for normally distributed variables, median (interquartile range) for non‐normally distributed variables, and frequency (percentage) for categorical variables. Baseline characteristics (e.g., age, BMI, disease duration) will be compared between the two groups to assess group equivalence. Given the two‐group design (intervention vs control) with four time points (baseline, mid‐intervention, post‐intervention, and follow‐up), a two‐way repeated‐measures ANOVA (group × time) will be used for normally distributed outcomes to evaluate main effects and interaction effects. For non‐normally distributed data, the Friedman test (within‐group) and Mann–Whitney *U* test (between‐group at each time point) will be applied, with appropriate post hoc analyses. Within‐group changes over time will be assessed using repeated‐measures ANOVA (or Friedman test), while between‐group differences at each time point will be examined using independent *t*‐tests (or Mann–Whitney *U* tests). When significant interactions are identified, post hoc pairwise comparisons with appropriate corrections (e.g., Bonferroni) will be performed. Effect sizes (e.g., partial eta squared or Cohen's *d*) and mean differences with 95% confidence intervals will be reported alongside p‐values. All tests will be two‐tailed, with statistical significance set at *p* < 0.05. An intention‐to‐treat (ITT) approach will be applied, and missing data will be handled using the last observation carried forward (LOCF) method. Additionally, change scores across all four time points will be calculated to support the interpretation of clinical relevance.

### 2.14 Safety Measures and Adverse Effects Management

Although major side effects are unlikely, participants will be closely monitored throughout the intervention. Any adverse events (e.g., seizure, skin burn) will result in immediate discontinuation of FES and will be reported to the ethics board within 24 h. Participants will be informed of potential mild side effects such as pain, skin irritation, or discomfort, and advised to report them promptly. A history of epilepsy or seizures will be screened prior to FES application. Any serious adverse events or protocol modifications will be documented and submitted to the Institutional Review Board. Safety outcomes will be included in the final trial report to ensure comprehensive risk–benefit evaluation.

### 2.15 Ethics and Dissemination

This trial has been approved by the ethical permission from the ethics committee of the Institutional Review Board of Physiotherapy and Rehabilitation Department, Jashore University of Science and Technology, Jashore, Bangladesh (PTR‐JUST/IRB/2024/09/Project 0032) on September 21, 2024. Also, the trial is registered with the Clinical Trials Registry‐India (CTRI), the primary trial site of the World Health Organization, as CTRI/2025/07/091676 [Registered on: 24/07/2025]; Trial Registered Prospectively. The study must adhere to the Declaration of Helsinki as per the ethical guidelines. All participants will provide written informed consent before enrollment in this study and may withdraw at any time without issue. The trial's participants will be assured that their usual treatment regimen will not be affected by their participation in or withdrawal from the study. The findings will be published in peer‐reviewed journals. Additionally, findings will be presented at conferences around the country and the world.

### 2.16 Current Status of the Trial

The trial has not yet recruited participants.

## 3 Discussion

Despite rehabilitation, many stroke survivors continue to experience upper extremity impairments that significantly affect their activities of daily living (ADLs). This study will examine the application of FES‐integrated UPT to enhance upper‐extremity mobility in individuals who have experienced a stroke, with follow‐up to assess long‐term effectiveness. These findings may contribute to the development of evidence‐based intervention strategies for stroke rehabilitation in Bangladesh and other settings. Consequently, it could have significant implications for rehabilitation practices in low‐ and middle‐income countries. A key strength of the study is its randomized treatment allocation, which minimizes selection bias and strengthens the validity of the results. Stroke patients with severe pain may have impaired hand function. Hand function in stroke patients is linked to discomfort, deficits, and physical function. Pharmaceutical management has limited effects, while non‐pharmacological therapies, including exercise therapy, have short‐term effects in stroke sufferers [37]. This study addresses a research gap by exploring how FES‐integrated exercise treatment improves hand pain and function in stroke survivors, with an emphasis on assessments of improvement with rigorous methodology. Several studies have evaluated the impact of FES on the hand function of stroke patients [38, 39], and most have also been poorly designed and have had inadequate sample sizes. These studies suggest that FES improves hand function. The current research also suggests that further investigation is necessary to determine the long‐term effects of integrating FES with physiotherapy on hand pain and function. Stroke patients with hand disability are treated in institutional care using a safe hand functional rehabilitation strategy [40]. The usual physiotherapy treatment in this study was developed based on consensus guidelines for stroke rehabilitation [41], the national protocol of the Australian Ministry of Health [42], and evidence from high‐quality contemporary stroke literature [43]. These interventions were selected to ensure equal care for both groups. The study will be conducted at a renowned Bangladeshi physiotherapy and rehabilitation facility that uses evidence‐based therapeutic protocols. It aims to evaluate the comparative effectiveness of FES combined with usual physiotherapy treatment versus placebo FES with usual physiotherapy in improving hand pain and upper extremity function among stroke survivors. Following the intervention, all participants will receive free exercise guidance, and the study will adhere to SPIRIT and CONSORT standards, incorporating appropriate blinding and masking. The study will employ valid and reliable outcome measures and implement procedures to prevent cross‐contamination of data. Since the questionnaire is not available in Bengali, the study used standard translation. The project will also train trial stakeholders. Despite its complexity, the study process is meticulously designed, synchronized, and purposeful. This study will briefly discuss the paradigm shift in stroke treatment and the prospective use of FES. This study's strengths include addressing a recognized research gap, adherence to established protocols and guidelines, and careful planning to ensure an adequate sample size, calculated using G*Power to achieve sufficient statistical power. Simple random sampling ensured unbiased group allocation, valid outcome measures will be used, and rigorous blinding and masking procedures will be applied to maintain methodological rigor. This study may have some limitations, including its exclusive focus on Bangladesh, variability in patients' comorbidities, diversity in patients' clinical conditions and stroke severity, and a broad age range, which may affect the overall findings. Despite these constraints, the findings from the rigorously designed randomized controlled trial will provide valuable insights into stroke rehabilitation. Future research may investigate the effectiveness of FES compared with no electrical stimulation, or when combined with more specific or structured rehabilitation programs, using larger sample sizes and longer follow‐up periods.

## Author Contributions

**Ehsanur Rahman:** conceptualization, data curation, investigation, methodology, supervision, project administration, writing – original draft, writing – review and editing. **Saiba Muhammad Sabrin:** conceptualization, investigation, methodology, writing – original draft, writing – review and editing. **Md. Feroz Kabir:** conceptualization, formal analysis, writing – review and editing, validation. **Md. Obaidul Haque:** investigation, resources, supervision, project administration, writing – review and editing. **Md. Waliul Islam:** investigation, software, visualization, writing – review and editing. **Md. Zahid Hossain:** data curation, software, visualization, writing – review and editing, validation. **Md. Tofajjal Hossain:** resources, writing – review and editing. **Kazi Md. Azman Hossain:** data curation, investigation, methodology, writing – original draft, writing – review and editing. **Hassan M. Al‐ Emran:** conceptualization, formal analysis, supervision, funding acquisition, writing – review and editing.

## Ethics Statement

Ethical approval was obtained from the Institutional Review Board of the Physiotherapy and Rehabilitation Department, Jashore University of Science and Technology, Jashore, Bangladesh (PTR‐JUST/IRB/2024/09/Project 0032) on September 21, 2024. The study will follow the 2020 Declaration of Helsinki. All participants will provide written informed consent before enrollment in this study and may withdraw at any time without issue. Participant rights will also be protected in accordance with the World Medical Association's ethical guidelines.

## Consent

The authors have nothing to report.

## Conflicts of Interest

The authors declare no conflicts of interest.

## Transparency Statement

The lead author, Ehsanur Rahman, affirms that this manuscript is an honest, accurate, and transparent account of the study being reported; that no important aspects of the study have been omitted; and that any discrepancies from the study as planned (if relevant) have been explained.

## Data Availability

No data are available as no datasets were generated or analyzed for this study. Upon completion of this study, the participants' datasets and the statistical analysis will be made available to the corresponding author upon reasonable request.
